# Indiana Lunatic Asylum

**Published:** 1849-02

**Authors:** 


INDIANA LUNATIC ASYLUM.

We are glad to be able to communicate the following intelligence respecting the Indiana Hospital for the Insane, of which the report of the commissioners and superintendent has been published. We learn from these documents that the buildings are so far completed as to be able to accommodate about fifty patients. Preference is to be given to cases of recent origin, in which there is the greatest probability of effecting cures. When completed, about 200 patients can be accommodated. The cost of the building, when finished, is estimated at $72,059 22, or about $400 to each patient, which is below the average cost of similar institutions in other States. Dr. R. J. Patterson is the superintendent, and Mrs. Laura Ann Elliott the matron, both formerly of the Ohio Lunatic Asylum, and who are represented as well qualified for the discharge of the important duties assigned to them.

